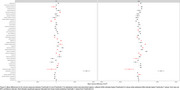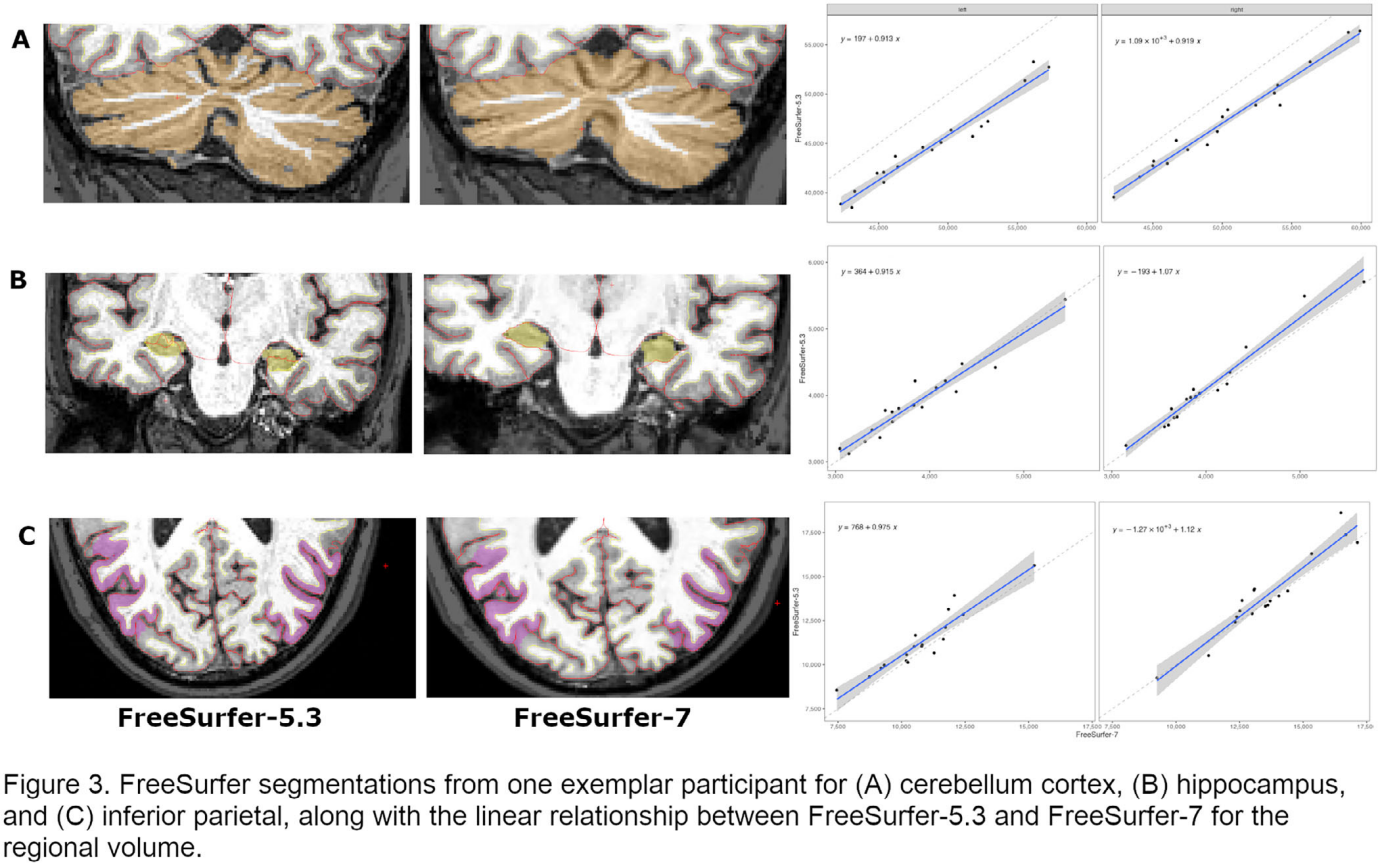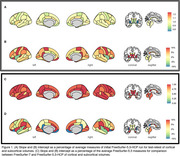# Variability in Volumetric Measures Between Different Versions of FreeSurfer

**DOI:** 10.1002/alz70856_106081

**Published:** 2026-01-08

**Authors:** Jacqueline Rizzo, Hope Shimony, Charles D. Chen, Sarah J. Keefe, Kristine E. Shady, Rebecca L. Feldman, Jalen Scott, Thomas Hunter Smith, Kaitlyn Dombrowski, Ashlee Simmons, John C. Morris, David M. Holtzman, Brian A. Gordon, Tammie L.S. Benzinger, Shaney Flores

**Affiliations:** ^1^ Washington University School of Medicine, St. Louis, MO, USA; ^2^ Massachusetts General Hospital, Harvard Medical School, Boston, MA, USA; ^3^ Washington University in St. Louis, St. Louis, MO, USA; ^4^ Knight Alzheimer Disease Research Center, St. Louis, MO, USA; ^5^ Washington University School of Medicine in St. Louis, St. Louis, MO, USA

## Abstract

**Background:**

FreeSurfer is a widely‐used program for segmenting cortical and subcortical regions of interest (ROIs) from magnetic resonance imaging (MRI) scans. Regional volumetric measures can be calculated from these ROIs and other imaging modalities may use the ROI segmentations for quantification. FreeSurfer has recently undergone several updates to improve performance. Here, we compare volumetric measures across different FreeSurfer versions.

**Method:**

Two T1‐weighted structural head MRI scans were acquired within the same session for 18 cognitively unimpaired participants (mean age: 63.7 years, ages: 48‐76 years, 14 females) with a Clinical Dementia RatingÒ of 0 on a Siemens 3T Trio MRI scanner. To examine test‐retest variability, slopes and intercepts were extracted from general linear models for regional volumetric measures after processing both scans through a Dockerized FreeSurfer‐5.3 container. For exploring inter‐version variability, the first T1 scan was processed through a Dockerized FreeSurfer‐7.4 container. Slopes and intercepts were extracted from general linear models predicting regional FreeSurfer‐7.4 measures from the associated 5.3 measures from the first T1 scan. Intercepts were assessed to determine if there was a significant offset in our test‐retest group and between FreeSurfer versions.

**Result:**

Volumetric measures for our test‐retest group showed significantly variable intercepts for two separate T1 scans. While our inter‐version comparison showed similarly strong linear association, inter‐version intercepts that removed the test‐retest offset were still significantly more variable for cortical and subcortical volumes (Figure 1). Some of the largest inter‐version intercept differences were observed for cerebellum white matter (left: t=3.77, *p* <.001; right: t=3.18, *p* <.001), parahippocampal (left: t=3.20, *p* <.001; right: t=4.78, *p* <.001), and right inferior parietal (t=2.50, *p* = .002) volumes (Figure 2 and Figure 3).

**Conclusion:**

Volumetric measures varied significantly between FreeSurfer‐5.3 and 7. Regions with the largest differences, such as cerebellum cortex, should be investigated for their impact on other imaging modalities, such as positron emission tomography, that use those regions for quantification.